# Management of urticaria in COVID‐19 patients: A systematic review

**DOI:** 10.1111/dth.14328

**Published:** 2020-10-09

**Authors:** Eyad Abuelgasim, Ann Christine Modaragamage Dona, Rajan Singh Sondh, Amer Harky

**Affiliations:** ^1^ Faculty of Medicine Imperial College London London UK; ^2^ St. George's Hospital Medical School University of London London UK; ^3^ Department of Cardiothoracic Surgery Liverpool Heart and Chest Hospital Liverpool UK; ^4^ Department of Integrative Biology, Faculty of Life Sciences University of Liverpool Liverpool UK; ^5^ Liverpool Centre for Cardiovascular Science Liverpool Heart and Chest Hospital Liverpool UK

**Keywords:** angioedema, antihistamines, COVID‐19, SARS‐CoV‐2, steroids, urticaria

## Abstract

The global pandemic COVID‐19 has resulted in significant global morbidity, mortality and increased healthcare demands. There is now emerging evidence of patients experiencing urticaria. We sought to systematically review current evidence, critique the literature, and present our findings. Allowing PRISMA guidelines, a comprehensive literature search was carried out with Medline, EMBASE, Scopus, Cochrane, and Google Scholar, using key MeSH words, which include “COVID‐19,” “Coronavirus,” “SARS‐Cov‐2,” “Urticaria,” “Angioedema,” and “Skin rash” up to 01 August 2020. The key inclusion criteria were articles that reported on urticaria and/or angioedema due to COVID‐19 infection and reported management and outcome. Studies were excluded if no case or cohort outcomes were observed. Our search returned 169 articles, 25 of which met inclusion criteria. All studies were case reports, reporting 26 patients with urticaria and/or angioedema, COVID‐19 infection and their management and/or response. ajority of patients (n = 16, 69%) were over 50 years old. However, urticaria in the younger ages was not uncommon, with reported case of 2 months old infant. Skin lesions resolved from less than 24 hours to up to 2 weeks following treatment with antihistamines and/or steroids. There have been no cases of recurrent urticaria or cases nonresponsive to steroids. Management of urticarial in COVID‐19 patients should involve antihistamines. Low dose prednisolone should be considered on an individualized basis. Further research is required in understanding urticarial pathogenesis in COVID‐19. This will aid early diagnostic assessment in patients with high index of suspicion and subsequent management in the acute phase.

## 1 INTRODUCTION

The global pandemic COVID‐19 is caused by severe acute respiratory syndrome coronavirus‐2 (SARS‐COV2). It has resulted in global morbidity, mortality and significantly increased healthcare demands.^1^, ^2^ It was originally reported that the main symptoms of COVID‐19 to be a cough and fever. However, as the pandemic progressed, our understanding of COVID‐19 increased, leading to anosmia and/or hyposmia established as a third symptom. As our understanding of this disease increases, it is reported that SARS‐COV2 can present with clinical manifestations beyond the respiratory system. We are now aware that neurological manifestation can develop which encompasses acute skeletal muscle injury as well as an impaired consciousness.^3^ Additionally, severe infections can have an impact on renal and cardiac function.^4^

More recently, there has been a growing interest regarding the dermatological manifestations in patients with COVID‐19. Skin manifestations during the course of a COVID‐19 infection was first reported in China, however the prevalence was low at 0.2% cases out of 1099 cases.^5^ There is now emerging evidence in literature making reference to some patients experiencing urticaria. Urticaria manifests itself as urticarial plaques that affect the upper dermis which can cover the skin and mucous membranes. It is described as erythematous and pruritic, and can sometimes present with angioedema, a type of swelling of the dermis subcutaneous tissue, the mucosa, and submucosal tissues.^6^

The objective of this systematic review is to review the current literature on urticaria in COVID‐19 patients. Furthermore, we aim to provide insight into urticarial pathogenesis and management in such patients.

## 2 METHODS

### 2.1 Literature search

This study was done according to Preferred Reporting Items for Systematic Reviews and Meta‐Analyses (PRISMA) method identifying published literature on urticaria and/or angioedema due to COVID‐19 infection and its management and outcomes. The comprehensive literature search was carried out with Medline, EMBASE, Scopus, Cochrane database, and Google Scholar, using key MeSH words, which include “COVID‐19,” “Coronavirus,” “SARS‐Cov‐2,” “Urticaria,” “Angioedema,” and “Skin rash.” Manual cross checking of reference lists of relevant articles was performed. All published articles have been reviewed, and the findings have been included in this study. The relevant articles have been cited and referenced within this study. The limits included studies in English and articles published after December 2019 until 01 August 2020. All the relevant articles identified were analyzed by two authors, and the results were appropriately summarized and reported.

### 2.2 Inclusion and exclusion criteria

The key inclusion criteria were articles that reported on urticaria and/or angioedema due to COVID‐19 infection and reported management and outcome, and studies were excluded if no case or cohort outcomes were observed. Other exclusion criteria were consensus documents, editorials, commentaries, and narrative reviews.

### 2.3 Data extraction

All studies were screened by two authors independently (E.A. and A.D); disagreement was resolved by consensus or involvement of other authors (R.S. and A.H.). The extracted data then were crosschecked by a third author to validate their accuracy (A.H.).

## 3 RESULTS

**FIGURE 1:**
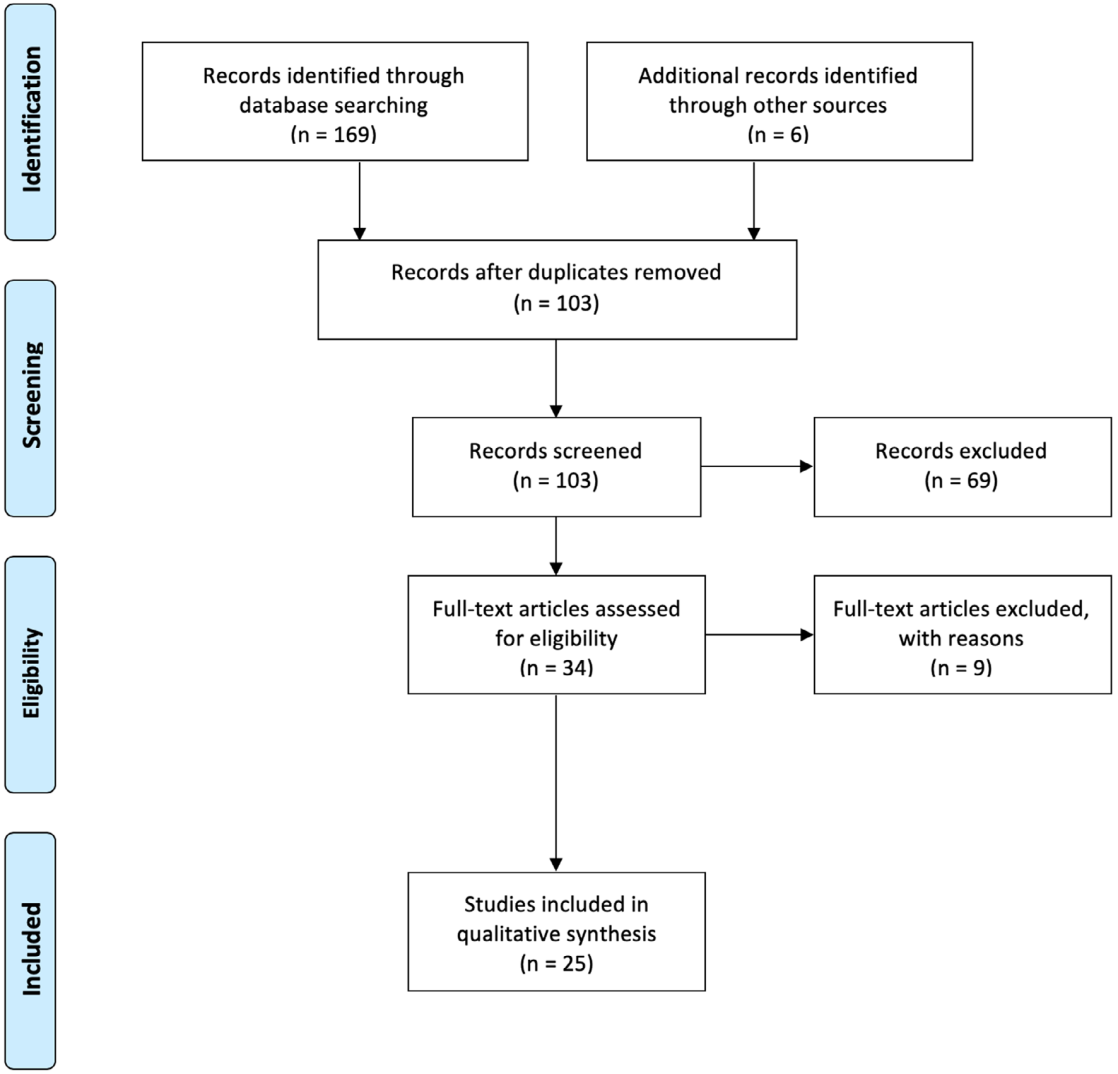
Article selection flowchart (PRISMA)

Following an extensive database search, 169 articles were identified. Of these, 34 were selected for full text review based on their title and abstract. Full text screening resulted in the final selection of 25 articles (Figure 1),^7^, ^8^, ^9^, ^10^, ^11^, ^12^, ^13^, ^14^, ^15^, ^16^, ^17^, ^18^, ^19^, ^20^, ^21^, ^22^, ^23^, ^24^, ^25^, ^26^ reporting 26 patients with urticaria and/or angioedema and COVID‐19 infection and their management plan and/or response to management. Table 1 includes the summarized key findings of the studies included in this review. All included articles were case reports.

**TABLE 1:** Management and response of patients with urticaria and/or angioedema during COVID‐19 infection

Study	Case characteristic	Cutaneous manifestation	Involvement site	Accompanied by COVID‐19 symptoms	Skin biopsy	Medical and drug History	Management	Response to management	Duration of skin lesions
Proietti et al^7^	6‐month‐old, male infant	Giant urticaria, with multiple lesion	Mainly affecting the trunk and limbs	Asymptomatic. 2 weeks after COVID‐19 confirmed by RT‐PCR	Not reported	Not correlated with drugs (topical or systemic), bacterial or parasitic infections, inhalant exposure, or insect bites. Allergies such as allergic rhinitis, atopic dermatitis, and food allergy were not reported.	Laboratory findings were within the normal ranges. Betamethasone (soluble tablets, 0.5 mg/day for 7 days)	Clinical improvement following treatment	<7 days
Sousa Gonçalves et al^8^	57‐year‐old Caucasian man	Urticarial rash (an erythematous papular rash with irregular contours	Elbows	6 days after first reporting COVID‐19 symptoms	Not reported	No newly initiated drugs, patient did not have atopy or a clinical history of allergy or other conditions	Not reported	Not reported	Not reported
Rolfo et al^9^	62‐year‐old current smoker man with diagnosed T4N2M1b G3 stage IV squamous cell lung carcinoma with pleuro‐pulmonary involvement	Urticarial papular lesions, with marked itching and minimal erythema	Lower dorsal, lumbar and gluteal region	2 days after first reporting COVID‐19 symptoms. Two days before COVID‐19 confirmed by RT‐PCR	Vasculitis involving the superficial and deep dermis, with signs of microangiothrombosis, showing fibrinoid changes of vessel wall with some granulomas, neutrophilic infiltrate, and nuclear debris.	2 days after the last immunotherapy dose—ipilimumab (1 mg/kg every 6 weeks) plus nivolumab (3 mg/kg every 2 weeks)	Serial ferritin, D‐Dimer (DD), and IL6 in addition to ANAS and C4, to discard differential diagnoses, were evaluated. Elevation of ferritin (940 ng/mL) and DD (2.600 ng/dL) was documented. Hydroxychloroquine (400 mg BID on day 1200 mg BID for 14 days). Azithromycin (500 mg day 1250 mg days 2–5) Methylprednisolone 1 mg/. kgEnoxaparin 40 mg SC/day	Within 14 days, dominant skin lesions disappeared, cough and chest CT‐scan normalized. ANAS and complement C4 normalized, as were clotting times and fibrinogen. Serial evaluation of IL6 levels by ELISA only had a slightly elevated value of 246 pg/mL (range 6.25‐200 pg/mL,) and throughout the 18‐day follow‐up period there was lymphopenia that became less evident	14 days
Shanshal^10^	31‐year‐old lady with a 5‐year history of well‐controlled chronic urticaria	Extensive, severely itching urticarial lesions	Mainly concentrated on the trunk and extremities and sparing of the face, palm, and sole	5 days after first reporting COVID‐19 symptoms.3 days before COVID‐19 confirmed by RT‐PCR	Not reported	Nonsedating antihistamines	Low‐dose systemic steroid and nonsedating antihistamine	Rash controlled within 5 days	5 days
Hassan^11^	46‐year‐old female nurse with history of hay fever and mild asthma	Widespread urticarial eruption; red‐raised blanching and itchy rash with angioedema of lips and hands	Face, arms, torso, legs, and loins	48 hours before developing COVID‐19 symptoms. 2 days before COVID‐19 confirmed by RT‐PCR	Not carried out	No prescribed regular medications no over‐the‐counter medications	Started fexofenadine hydrochloride 180 mg, two to four times per day. Rash worsened following day and was associated with angioedema. Advised to continue taking fexofenadine hydrochloride 180 mg four times per day and she was commenced on prednisolone 40 mg once daily for 3 days. Prednisolone helped lip and hand swelling, but rash remained itchy.Chlorphenamine maleate 4 mg four times/day was subsequently added.	The rash resolved completely over next few days. The patient made a full clinical recovery	Around 14 days
Najafzadeh et al^12^	Elderly man	Pruritic hives 1.5‐8.0 cm in diameter	Generalized urticaria with angioedema of face and neck	At same time as COVID‐19 symptoms	Not reported	Not reported	Initial biochemical tests showed low numbers of white blood cells (WBC) (WBC = 2.75 × 10^3^). Lymphopenia was detected (lymphocytes = 852). RT‐PCR for COVID‐19 was not performed. CT chest was carried out, which showed pneumonia with bilateral and subpleural areas of ground‐glass opacification, consolidation affecting the lower lobes and confirming the diagnosis of COVID‐19.	Not reported	Not reported
de Perosanz‐Lobo et al^13^	Elderly woman admitted to the hospital with bilateral pneumonia testing positive for COVID‐19	Painful erythematous patches which left residual purpura when fading	Trunk, buttocks, and hips	> 5 days after first reporting COVID‐19 symptoms	Histologic changes characteristic of small‐vessel urticarial.Vasculitis: blood extravasation and neutrophilic perivascular inflammation with prominent karyorrhexis. There are some macrophages with a cytoplasm full of nuclear debris	Treatment with hydroxychloroquine, lopinavir/ritonavir, and azithromycin for 5 days	A sudden worsening of respiratory condition led to the patient's death, and therefore, no treatment could be prescribed.	Mortality	N/A
	Middle‐aged man with a 14‐day history of fever, cough and anosmia	Erythematous and edematous plaques with active border and purpuric center	Buttocks	14 days after first reporting COVID‐19 symptoms	Evidence of small‐vessel damage: preserved epidermis with moderate perivascular neutrophilic inflammation and blood extravasation in the dermis. Endothelial swelling, necrosis and fibrin deposition	Not reported	Therapy with hydroxychloroquine and azithromycin was started as treatment for COVID‐19. Prednisone and antihistamines were administered for his skin condition.	14 days later, the patient was asymptomatic.	14 days
Falkenhain‐López et al^14^	51‐year‐old otherwise healthy woman with a 3‐day history of dry cough and arthralgias	Widespread pruritic evanescent skin lesions (lasting <24 hours).Multiple well‐demarcated erythematous edematous papules and plaques with diffuse underlying erythema	Trunk, thighs, upper limbs, and predominantly on the facial area and dorsal aspects of bilateral hands	3 days after first reported COVID‐19 symptoms and confirmation of COVID‐19 by RT‐PCR		The patient had not taken any medication before the onset of the symptoms.No recent contact with plants, chemicals, or topical products. No urticarial lesions before, and no precipitating factors were found.Review of systems was negative for diarrhea, dysphagia, or other suggestive symptoms of anaphylaxis.	Blood test showed lymphopenia and elevated C‐reactive protein (5.4 mg/L) and LDH (388 U/L). Chest radiography revealed bilateral pulmonary infiltrates. Treatment with loratadine 10 mg every 12 hours	Early improvement of pruritus and resolution of skin lesions within 2 days.The patient did not experience recurrent episodes of urticaria after 7 days of antihistaminic treatment.	7 days
Goldust et al^15^	74‐year‐old Wuhan man presented with fever (100.4 F), dry cough and fatigue	Diffuse, irregular shaped, partially confluent urticarial wheals	Generalized	12 days after admission, first reported COVID‐19 symptoms and confirmation of COVID‐19 by RT‐PCR	Not carried out	Treatment included hydroxychloroquine, lopinavir/ritonavir, thymosin, and methylprednisolone.	A CT scan of the lung showed ground‐glass changes. Treatment included hydroxychloroquine, lopinavir/ritonavir, thymosin, and methylprednisolone. (unclear which medications were started before/after development of urticaria—possible reaction to medication?)	Not reported	Not reported
	65‐year‐old subfebrile (98.6 F) Wuhan woman had dry cough, fatigue and diarrhea (four times a day)	Disseminated, variable size, erythematous patches, which fade on pressure. Few patches were confluent.	Generalized	1 day after admission	Not carried out	Ruxolitinib	CT scan showed bilateral ground‐glass changes. RT‐PCR swabs did not detect SAR‐Cov‐2.Symptoms considered as unspecific viral rash due to COVID‐19 and included as differential diagnosis a drug eruption due to the antineoplastic drug ruxolitinib.	Not reported	Not reported
Aktaş et al^16^	64‐year‐old female	Severe pink urticarial plaques	Generalized	During course of COVID‐19	Not reported	Metformin and a combination of irbesartan and hydrochlorothiazide treatment for years due to diabetes mellitus and hypertension. No atopy in dermatological examination. Similar reaction occurred 9 years ago lasting a few weeks.	Detailed investigation including thorax computed tomography and testing coronavirus. Treated with hydroxychloroquine, azithromycin, and oseltamivir in intensive care unit for 7 days. As etiology of her diffuse urticaria, viral infection itself, drugs she received, and psychological stress of the clinical condition were considered. Cetirizine 10 mg twice a day.	Urticarial reaction was partially controlled on Cetirizine 10 mg twice a day	Not reported
Diotallevi et al^17^	55‐year‐old woman admitted for pyrexia, dry cough, and dyspnea	Urticarial skin rash characterized by erythematous, smooth, slightly elevated papules and wheals, associated to severe pruritus.	Generalized	3 days before admission and confirmation of COVID‐19 by RT‐PCR	Not reported	No new medication before the rash appeared. The patient did not report neither similar episodes in the past, nor allergies to drugs or foods.	High‐resolution computed tomography scan of the chest revealed a diffuse bilateral ground‐glass opacity. Blood test revealed normal blood count (no lymphopenia or lymphocytosis or eosinophilia), slight increase of procalcitonin serum level (0.14 ng/mL), C‐reactive protein (CRP, 12.1 mg/dL), and liver enzymes (GOT, GPT, LDH, GGT fourfold levels). A systemic treatment with intravenous daily administration of betamethasone sodium phosphate 4 mg and chlorphenamine maleate 10 mg, in addition to antiviral therapy with lopinavir/ritonavir for pneumonia	In the following days urticaria improved gradually.Twenty‐five days after admission, patient was discharged.	Not reported
	64‐year‐old patient with acute respiratory distress syndrome (PaO^2^/FiO^2^ ≤ 100 mm Hg) caused by COVID‐19	Urticarial rash	Generalized	Skin rash was already present at the time of hospital admission	Not reported	Treatment with lopinavir/ritonavir and hydroxychloroquine from 1 week, and no new drug introduction had been made in the last 3 weeks before skin rash development. No history of allergy to drugs or foods, nor recent intake of new drugs	Blood test revealed abnormal blood count with neutrophil leukocytosis (neutrophil granulocytes 8.600/mm^3^), and mild lymphopenia (lymphocytes 700/mm^3^), moderate increase of pro‐calcitonin serum levels (0.87 ng/mL), marked increase of CRP (10.2 mg/dL), and liver enzymes (GOT, GPT, LDH, GGT fourfold levels) serum levels. Mechanical ventilation for respiratory failure. Intravenous administration of methylprednisolone 40 mg/die and bilastine 20 mg/die.	Skin rash is slightly improved after 48 hours from the beginning of the treatment. Patient in stable condition.	Not reported
de Medeiros et al^18^	55 years old female, intensive care physician	First episode: Painful erythematous‐edematous plaques.Some lesions evolved into bruises. Second episode: Exuberant urticarial lesions. Light erythema and edema with intense itching	First episode: Flexor face of forearms and leg extensors|. Second episode: Exuberant urticarial lesions on shoulders. and inguinal region. Light erythema and edema on palms	First episode: 5 days after contact with COVID‐19 ICU patient. Second episode: 2 days after second exposure with COVID‐19 ICU patient. At same time as COVID‐19 symptoms	Not reported	Not reported	First episode: Betamethasone cream 0.1% once a day. Second episode: Bilastine 20 mg one tablet a day for 15 days.Betamethasone ointment 0.1% cream once a day for 2 daysConfirmation of COVID‐19 by RT‐PCR.	First episode: lesion resolution in 3 days. Second episode: Within 48 hours, there were no more wheals and erythematous‐edematous plaques appeared without itching in the antecubital and popliteal fossae.Lesions regressed after the use of betamethasone	First episode: 3 days. Second episode: 4 days
Cepeda‐Valdes et al^19^	Patient 1 was a 50‐year‐old woman, and Patient 2 was a 20‐year‐old woman, who was the daughter of Patient 1	Bilateral disseminated rash characterized by erythematous annular and irregular wheals on the skin that appeared suddenly and disappeared within <24 hours	Shoulders, elbows, knees, and buttocks	After developing COVID‐19 symptoms	Not reported	Neither patient had any history of similar lesions, and no trigger factors other than the viral context were identified	Antihistamines and moisturizers	48 hours after treatment was started the urticaria resolved	2 days
Naziroğlu et al^20^	53‐year‐old male	Pruritic edematous plaques	Generalized	No respiratory or systemic symptoms	Not reported	No previous history of atopic conditions including drug or food allergy, chronic urticaria.	Treatment was started with diagnosis of COVID‐19	On the fourth day of his admission, his skin lesions regressed and he was discharged on the fifth day of his admission	4 days
Gunawan et al^21^	51‐years‐old male	Pruritic urticaria		On day 3 of hospitalization, after presenting with COVID‐19 symptoms	Not reported	History of hypertension, diabetes, dyslipidemia and hyperuricemia on therapy. No urticaria triggers other than viral infection were found, as there was no history of food allergy, drug allergy, chronic urticaria, or other allergies. There was no history of consuming new medicine in 15 days prior besides COVID‐19 treatment in hospital.	Patient was treated with azithromycin, hydroxychloroquine, cefoperazone‐sulbactam, omeprazole, and medicines for his comorbidities. Oral antihistamine loratadine was added to his treatment with improvement of symptom on the next day. The suspicion of urticaria caused by the medicines given in hospital could be eliminated by the fact his urticaria improved even the medicines continued to be given.	Improvement of symptom on the next day	24 hours
Adeliño et al^22^	30‐year‐old female physician	Rapidly spreading wheals.In a few hours, face wheals promptly converted to facial angioedema, with preferential involvement of periocular region and mild edema of the lips, without compromise of the tongue, uvula, vocal cords, or the airway.	Face, trunk, abdomen, and limbs	On day +11 of disease evolution, after resolution of previous COVID‐19 symptoms	Not reported	No relevant past medical history except for pine seeds allergy, following a strict nut‐free diet since she was diagnosed. Family history of hereditary angioedema. Not on any medication. She had not taken nonsteroidal inflammatory drugs or angiotensin‐converting enzyme inhibitors the previous 15 days.She had not exercised, had not drunk alcohol, nor was on menstrual period.	Oral antihistamine (ebastine 10 mg *ter in die*)	24 hours after the onset of the cutaneous symptoms, both the wheals and angioedema started to fade off, turning into erythematous macules until complete resolution.	24 hours
Paolino et al^23^	37‐year‐old Caucasian woman, in her 10th postpartum day	Craniocaudal cutaneous manifestation characterized by erythematous maculopapular lesions	Trunk, neck, and face	3 days after first reporting COVID‐19 symptoms	Not reported	Acetaminophen	No signs of dyspnea, and the vital signs (including saturation) were all in normal range. A symptomatic treatment with only acetaminophen was prescribed seventh postpartum day prior development of rash. Breastfeeding has not been suspended.	After 8 days, the cutaneous lesions clearly improved along with improvement of the general symptoms and absence of fever and dry cough. The newborn did not show any symptom of the disease and did not develop any cutaneous lesion.	8 days
Ahouach et al^24^	57‐year‐old woman	Diffuse fixed erythematous blanching maculopapular lesions	Asymptomatic over the limbs and trunk, with burning sensation over the palms	48 hours before COVID‐19 symptoms	Slight spongiosis, basal cell vacuolation and mild perivascular lymphocytic infiltrate	No drug intake, except paracetamol for fever	No treatment	Fever and rash resolved within 9 days, dry cough within 2 weeks.	9 days
Quintana‐Castanedo et al^25^	61‐year‐old male physician	Progressive, mildly itchy urticarial rash consisting of confluent, edematous and erythematous papules	Thighs, arms and forearms. Palms and soles were spared.	Not reported	Not reported	No drug during last 2 months	Oral antihistamines	Remained afebrile during the next week. Cutaneous rash resolved in 7 days.	7 days
Rivera‐Oyola et al^26^	60‐year‐old woman	Sudden‐onset mild hemi‐facial atrophy and scoliosis, generalized, pruritic rash, large, disseminated, and urticarial plaques	Trunk, head, upper, and lower extremities	9 days after first reporting COVID‐19 symptoms	Not reported	Estradiol, for many months and allergy to propofol. No recent changes to her medications.	Fexofenadine	The patient recovered from her infection without sequelae and did not require hospitalization. Urticarial lesions did not recur on her discontinuation of the fexofenadine 1 week after starting.	1 day
Morey‐Olivé et al^27^	2‐month‐old girl	Acute urticaria, apparently pruritic	Face and upper extremities which spread in a few hours to trunk and lower extremities. The palms and soles were not affected.	4 days after low fever, at the same time with COVID‐19 symptoms	Not reported	Not reported	Oral symptomatic treatment	Most lesions healed within 24 hours, and the cutaneous manifestations resolved in 5 days in the absence of any other signs and symptoms	5 days
Amatore et al^28^	39‐year‐old male	Erythematous, rash, edematous nonpruritic annular fixed plaques of various diameters	Upper limbs, chest, neck, abdomen and palms, sparing the face, and mucous membranes	At same time as COVID‐19 symptoms	Histological findings were unspecific, consistent with viral exanthemata: superficial perivascular lymphocytic infiltrate, papillary dermal edema, mild spongiosis, lichenoid and vacuolar interface dermatitis, dyskeratotic basilar eratinocytes, occasional neutrophils but no eosinophils within the dermal infiltrate.	No relevant medical history. Taken no medications in the days and weeks prior to onset of symptoms	Oral hydroxychloroquine sulfate 200 mg three times per day for 10 days	No pulmonary symptoms developed.Rash fully recovered on day 6 of treatment	6 days
van Damme et al^29^	39‐year‐old female nurse	Pruritic urticarial rash	Generalized	At same time as COVID‐19 symptoms	Not reported	No change in her daily habits or drugs	Bilastine	Gradual improvement of rash	Not reported
Henry et al^30^	27‐year‐old woman	Pruritic rash, large, disseminated, and urticarial plaques	Particular face and acral involvement	48 hours before COVID‐19 symptoms	Not reported	No triggers except for the viral context were found, and common viral serology was negative.	Paracetamol and oral antihistamines	Slow improvement symptoms	Not reported
Cohen et al^31^	62‐year‐old man with a history of hypertension	12 hours of slightly asymmetric, and nonpitting edema of cheeks and lips	Lip and facial swelling.He had no other sites of swelling and had no rash.	12 days before COVID‐19 symptoms	N/A	Lisinopril	Leukocytosis with relative lymphopenia and elevated high‐sensitivity C‐reactive protein and D‐dimer. Functional C1 inhibitor levels (59.7 mg/dL), C3 levels (206 mg/dL), and C4 levels (46 mg/dL) were all elevated. Intravenous methylprednisolone, famotidine, and diphenhydramine. His lisinopril was held.	By hospital day 2, swelling markedly improved.Discharged home in stable condition.	2 days

The majority of patients (n = 16, 69%) were over 50 years old. However, urticaria in the younger ages was not uncommon, with reported case of 2 months old girl. Skin lesions were reported resolve from less than 24 hours to up to 2 weeks following treatment with antihistamines and/or steroids. There have been no cases of recurrent urticaria or cases nonresponsive to steroids.

## 4 DISCUSSION

### 4.1 Demographic of COVID‐19 patients with urticaria development

The review population revealed that the majority of patients (18 patients) affected by urticaria were over 50 years old. However, urticaria in the younger ages was not uncommon. Typically, urticaria has a peak onset of 20‐40 years and affects females more than males, which was found to be the case in this review. Lifetime incidence of urticaria is reported to be 15%.^32^ It has been reported that urticaria may be a rare manifestation of COVID‐19, which has been observed in just under 4% of COVID‐19 patients.^33^

Of note, most case reports have found skin manifestations to not be associated with disease severity^29^, ^33^ Conversely, a prospective Spanish cohort study reported that the presentation of urticaria and maculopapular skin lesions were associated with higher morbidity (severe COVID‐19 illness) and higher mortality rate (2%).^34^ Further observational studies will aid further understanding of the association of COVID‐19 disease progression and dermatological manifestations.

### 4.2 Pathophysiology of urticaria in COVID‐19

The pathophysiology was previously hypothesized to be attributed to drug‐induced urticaria. Urticaria is a well‐known cutaneous manifestation of a drug eruption,^35^ however, urticaria has been debated in COVID‐19 patients as to whether the virus directly results in urticaria, or if urticaria is caused by a drug eruption. There have been reports of COVID‐19 positive cases with urticaria, where there had been no changes in their medication regime.^26^, ^33^ This may suggest that urticaria could be directly related to the pathogenesis of the SARS‐CoV2. However, individual case reports have reported urticaria manifestation prior to commencement of therapy for COVID‐19 as well as reports of remission from urticaria despite continuation of drug therapy.^29^ This suggests that urticaria in COVID‐19 is likely multifactorial and drug‐associated skin manifestations to not account for all cases.

SARS‐CoV‐2 entry into a cell is mediated through binding to angiotensin‐converting enzyme‐2 (ACE2) protein and subsequent endocytosis in epithelial targets in the lung.^36^ Of note, systemic response may be owed to the presentation of ACE2 on other tissues, including kidney, brain and importantly, the vasculature. Angiotensin (Ang) I and Ang II are deactivated by ACE2 Ang I and Ang II are associated with inflammation, oxidative stress and fibrotic scarring.^37^ In the instance of coronavirus infection, the binding of SARS‐CoV‐2 with ACE2 disrupts normal ACE2 activity. This may result in increased activity of Ang II, leading to formation of reactive oxygen species, disrupt antioxidant and vasodilatory molecules, and result in complement activation.^38^ Such disrupted physiological processes were observed in a rat model with aberrant expression of Ang II.^39^

COVID‐19 associated skin manifestations may be mediated by the systemic inflammatory response that follows the human body's response to an acute infection.^40^ This includes activation of the complement system and adjustment of the cytokine‐chemokine milieu.^10^ Consequently, this progresses to aberrant activation and sequential degranulation of mast cells. It is hypothesized that mast cell degranulation is the principal pathophysiology associated with subsequent systemic organ damage in COVID‐19.^41^ Of note, most patients with COVID‐19 were reported to have elevated levels of circulating interleukin‐6 (IL‐6).^42^ Furthermore, colocalization of SARS‐CoV‐2 glycoproteins and respective complement mediators have been reported in peripheral cutaneous blood vessels.^43^ Therefore, it is possible that these mediators may be attributed to urticarial pathogenesis.

Urticaria has sometimes been associated with eosinophilia (>500 eosinophils/mm^3^), which has been observed in a number of COVID‐19 cases.^44^ Moreover, eosinophilia seems to have a protective mechanism and has been associated with a better prognosis.^45^ There have also been some cases where patients initially presented with urticaria only before experiencing the typical COVID‐19 symptoms and testing positive. What was evident in these cases was that they had been taking some form of prescribed medication prior to testing positive to COVID‐19.^46^, ^47^ Despite some patients having no medication changes, they still were taking medication at the time of onset of urticaria, suggesting that COVID‐19 may cause eosinophilia, resulting in drug hypersensitivity and thus urticaria. However, more research is needed to formally establish this relation.

### 4.3 Diagnosis assessment

It is important to ensure that urticaria is correctly diagnosed so that appropriate treatment can be administered. A diagnostic characteristic of urticaria is that the cutaneous lesions must be evanescent. Multiple case reports have not detailed this characteristic in their studies, so it is important this is taken into consideration. Furthermore, some case reports have mentioned how a skin biopsy for histopathological studies may aid in a diagnosis of urticaria.^48^ One case report has discussed that a skin biopsy of a COVID‐19 patient with urticaria revealed perivascular infiltrate of lymphocytes, some eosinophils and upper dermal oedema.^49^ A skin biopsy and awareness of evanescent lesions may allow for the differentiation to be made between urticaria and other cutaneous manifestations, limiting the chance of a misdiagnosis.

On clinical assessment clinicians should consider the possibility of glucose‐6‐pyruvate dehydrogenase (G6PD) deficiency in COVID‐19 patients as this group of patients may have a dominance of high‐producing IL‐6 allele. In one study group, this correlation has been reported in 71% of patients.^50^

### 4.4 Patient management

Classically, the recommended algorithm for treating urticaria includes the use of second‐generation antihistamines, and if inadequate control within 2‐4 weeks, the dose can be increased up to four times the original dose. If this is still inadequate control after a further 2‐4 weeks, specialist referral should be considered, where specialists can consider prescribing omalizumab and ciclosporin to help alleviate symptoms.^51^ However, in most patients, second generation oral antihistamines provide adequate control of urticaria.^52^ The pathophysiology of COVID‐19 related urticaria demonstrates that antihistamines alone will not stop mast cell histamine degranulation but will only act to reduce the severity of urticaria.

Low systemic steroids, on the other hand, target the COVID‐19 inflammatory storm, which prevents mast cell activation, and thus histamine release. Therefore, low dose systemic steroids may be able to effectively manage urticaria in COVID‐19 through their proposed mechanism of action. Combining this with antihistamines can improve patients' clinical response to urticaria^10^. A further benefit of low dose steroids, shown through a randomized control trial, has demonstrated an increase in survival rate in COVID‐19 patients (Randomized Evaluation of COVID‐19 Therapy [RECOVERY], ClinicalTrials.gov Identifier: NCT04381936). Although corticosteroids are promising, it may increase the risk of prolonged viral replication, so it may be best to use them for the shortest duration possible until symptoms are controlled. After this, consideration should be made to promptly switch to omalizumab. Ciclosporin is currently not recommended in COVID‐19 patients.^52^

### 4.5 Limitations

All included articles were case. Only three case reports detailed pathological study results.^9^, ^13^, ^28^ A diagnostic characteristic of urticaria is that the cutaneous lesions must be evanescent (no one lesion should last more than 24 hours), however this was only noted by Falkenhain‐López et al.^14^

## 5 CONCLUSION

Urticaria is a significant manifestation of COVID‐19, notably affecting patient morbidity. As such the clinical presentation of urticaria can aid diagnostic assessment, while considering risk factors, such as G6PD deficiency and aberrant IL‐6 expression. Management of COVID‐19 patients should involve antihistamines. Low dose prednisolone should be considered on an individualized basis. Further research is required in understanding urticarial pathogenesis in COVID‐19. This will aid early diagnostic assessment in patients with high index of suspicion and subsequent management in the acute phase.

## CONFLICT OF INTEREST

The authors declare no conflicts of interest.

## Data Availability

Data sharing is not applicable to this article as no new data were created or analyzed in this study.
